# SLFinder, a pipeline for the novel identification of splice-leader sequences: a good enough solution for a complex problem

**DOI:** 10.1186/s12859-020-03610-6

**Published:** 2020-07-08

**Authors:** Javier Calvelo, Hernán Juan, Héctor Musto, Uriel Koziol, Andrés Iriarte

**Affiliations:** 1 Laboratorio de Biología Computacional, Departamento de Desarrollo Biotecnológico, Instituto de Higiene, Facultad de Medicina, Universidad de la República, Montevideo, Uruguay; 2 Unidad de Genómica Evolutiva, Facultad de Ciencias, Universidad de la República, Montevideo, Uruguay; 3 Sección Biología Celular, Facultad de Ciencias, Universidad de la República, Montevideo, Uruguay

**Keywords:** SL trans-splicing, De novo assembly, RNAseq data

## Abstract

**Background:**

Spliced Leader trans-splicing is an important mechanism for the maturation of mRNAs in several lineages of eukaryotes, including several groups of parasites of great medical and economic importance. Nevertheless, its study across the tree of life is severely hindered by the problem of identifying the SL sequences that are being trans-spliced.

**Results:**

In this paper we present SLFinder, a four-step pipeline meant to identify de novo candidate SL sequences making very few assumptions regarding the SL sequence properties. The pipeline takes transcriptomic de novo assemblies and a reference genome as input and allows the user intervention on several points to account for unexpected features of the dataset. The strategy and its implementation were tested on real RNAseq data from species with and without SL Trans-Splicing.

**Conclusions:**

SLFinder is capable to identify SL candidates with good precision in a reasonable amount of time. It is especially suitable for species with unknown SL sequences, generating candidate sequences for further refining and experimental validation.

## Background

Spliced Leader (SL) trans-splicing, that is, the incorporation of a short RNA (the spliced leader) on the 5′ end of a different transcript, is an important but poorly understood part of the mRNA maturation process of many eukaryotic lineages. SL genes are often encoded in tandem repeats measuring a few kilobases, close to 5S rRNA genes [1] but there are exceptions (e.g. [2]). SL transcript sequences can be divided into two regions: an exon like sequence that remains in the final trans-spliced transcript and an intron that usually contains a canonical Sm-protein-binding site (see for exceptions: [3, 4]), separated by a splice donor site [1].

While there is no clear pattern with regards to specific metabolic pathways or functions for the transcripts that are subject to these mechanisms [5–7], SL trans-splicing participates in important regulatory functions such as operon resolution, 5’UTR edition and the incorporation of modified 5′ cap [1, 5, 6, 8, 9]. At least in some cases, it has been shown that it can also play an important role generating different isoforms by facultative SL trans-splicing (e.g. [10]) or alternative SL trans-splicing acceptor sites [11]. The number of classes of SLs (i.e., Spliced Leaders with a distinct sequence) and the number of copies in each genome varies among different organisms, and at least in some cases, there is evidence of specialization. For example, *Caenorhabditis elegans* has two distinct types of SLs, one (SL-1) is incorporated at the start of the operon and the other (SL-2) is used to resolve the downstream coding sequences into different transcripts [8]. In the planarian *Schmidtea mediterranea* it has been described one particular SL that is expressed preferentially on stem cells [12].

The molecular mechanisms involved are poorly understood and are subject of continuous research (e.g. [13]) but evidence indicates that it’s closely related to cis-splicing, with several shared regulatory signals [1, 14]. All identified SL transcripts share a similar secondary structure to the snRNAs (i.e., U1, U2, U4, and U5) that form the spliceosome, suggesting a common evolutionary history [1, 5, 14]. However, its evolution is a topic of debate among researchers, mainly due to the uneven distribution of SL Trans-Splicing across the phylogeny of eukaryotes [1, 5, 14].

So far SL Trans-Splicing has been reported in groups such as Euglenozoa [15, 16], Platyhelminthes [17, 18], Nematoda [19, 20], Urochordata [21], Rotifera [22], Cnidaria [23], Dinoflagellata [24], Crustaceans [25] and Amoebozoa [4]. However, it is absent in others such as vertebrates, insects, plants, Fungi and several protists [14, 26]. This brings the question if the mechanism has independently evolved several times (i.e., by modification of cis-splicing) or was present on the eukaryotic last common ancestor and lost many times [1, 5, 14, 25], with the discussion going back and forth as the mechanism is identified in new taxonomic groups (e.g. [25]).

When analyzing a new organism, the first obvious step is the identification of potential SL sequences on the mRNAs. This does not only allow to identify the presence of the mechanism in the group but having these sequences opens the possibility to use methodologies tailored toward SL Trans-Spliced transcripts. For example, “SL Trapping” [27] or “SL-seq” [28], both modified Next Generation Sequencing (NGS) protocols, allow an enriched sequencing of SL trans-spliced transcripts (e.g. [11]). Other approaches exist, but they either focus on identifying trans-splicing acceptor sites on the coding genes (e.g. [29, 30]), then requiring to be experimentally validated and providing no information about the specific SLs involved; or they require known SL sequences [31–34].

Unfortunately, the identification of SL sequences can be a significant roadblock due to technical limitations, specifically the reduced coverage of reads toward the transcript 5′ end that is typical of poly-A capture [35]. Combined with low or null sequence conservation across different phyla [5], within phylum variability, and several species with multiple SL classes with high nucleotide diversity [31, 36–38], these difficulties make the identification of SL sequences in new species a non-trivial problem. Several authors have tested different approaches to this problem with different degrees of automatization and reliance on previously known information (e.g. [5, 25, 31, 39–44]). Nevertheless, currently, there is no standardized protocol or analysis pipeline that allows the identification of putative SL (pSL) sequences, that is why often novel SL sequences are discovered almost by chance (e.g. [31]).

Here we present SLFinder, a four-step pipeline implemented in bash designed to facilitate the identification of novel SL exonic sequences from standard NGS RNAseq data (mRNA enriched by poly-A capture following a non-strand specific protocol). The pipeline first limits the potential candidates and provides a unifying command-line environment where parameters can be quickly adjusted to fit each species and dataset characteristics; while making limited assumptions on the SL sequence and mechanism, namely: 1) the SL sequence is located in the 5′ end of the transcript, 2) the SL sequence is present on the transcripts of many genes, 3) The sequence is not a palindrome, 4) There is at most one copy of it on each transcript, and 5) When mapped to the genome there is a canonical splicing donor site after the 3′ end (GT). In addition, and despite its limitations, the analyses are designed with transcriptome sequences generated using the widely used poly-A capture protocol so it can be applied to a larger group of organisms.

In order to evaluate SLFinder, we analyzed RNAseq data from several species with and without known SL Trans-Splicing and compared our predictions with the reported sequences in the bibliography. To better represent the intended use of the software on the identification of novel SL sequences, no manual intervention was carried out to curate the results (contrary to our recommendations when using this software).

### Implementation

#### Mandatory input data

For these analyses three inputs are necessary: 1) one or more assembled transcriptomes from the species of interest, following a de novo approach with Trinity [45, 46]; 2) a reference genome from the species and 3) an external database with Protein or cDNA (ideally from the same species or a reliable database such as SwissProt from Uniprot) for loci annotation.

Trinity can be replaced as the assembler following the instructions included on the manual, however, it is important to conduct an entirely de novo strategy to ensure that reads containing the SL sequence are not excluded of the final transcript. In addition, while we didn’t thoroughly test its effect, read normalization based on kmer frequency (e.g. [46]) is discouraged since reads from multiple transcripts will have the SL sequence and could potentially be partially discarded in some datasets. The longer the species SL sequences, the greater this issue is expected to be.

#### Strategy

Basically, the pipeline recovers potential SL exonic 3′ regions by looking for frequent kmers on the transcripts ends, extends them as much as possible by attempting to assemble them in contigs, and then filters out likely false positives based on sequence orientation, abundance, genomic data and overlap with annotation to known proteins. In practice, however, there are two issues to solve to implement such a straightforward approach. First, false positives due to biased kmer counts, which can be a result of the reconstruction of more than one isoform for a gene and other biological factors such as very similar transcripts from different genes of the same multigenic family. Second, the loss of strand information during sequencing in standard RNAseq sequencing, so that each transcript can be assembled either as the sense strand or as its reverse complement. Both main issues are addressed by our pipeline.

**Fig. 1:**
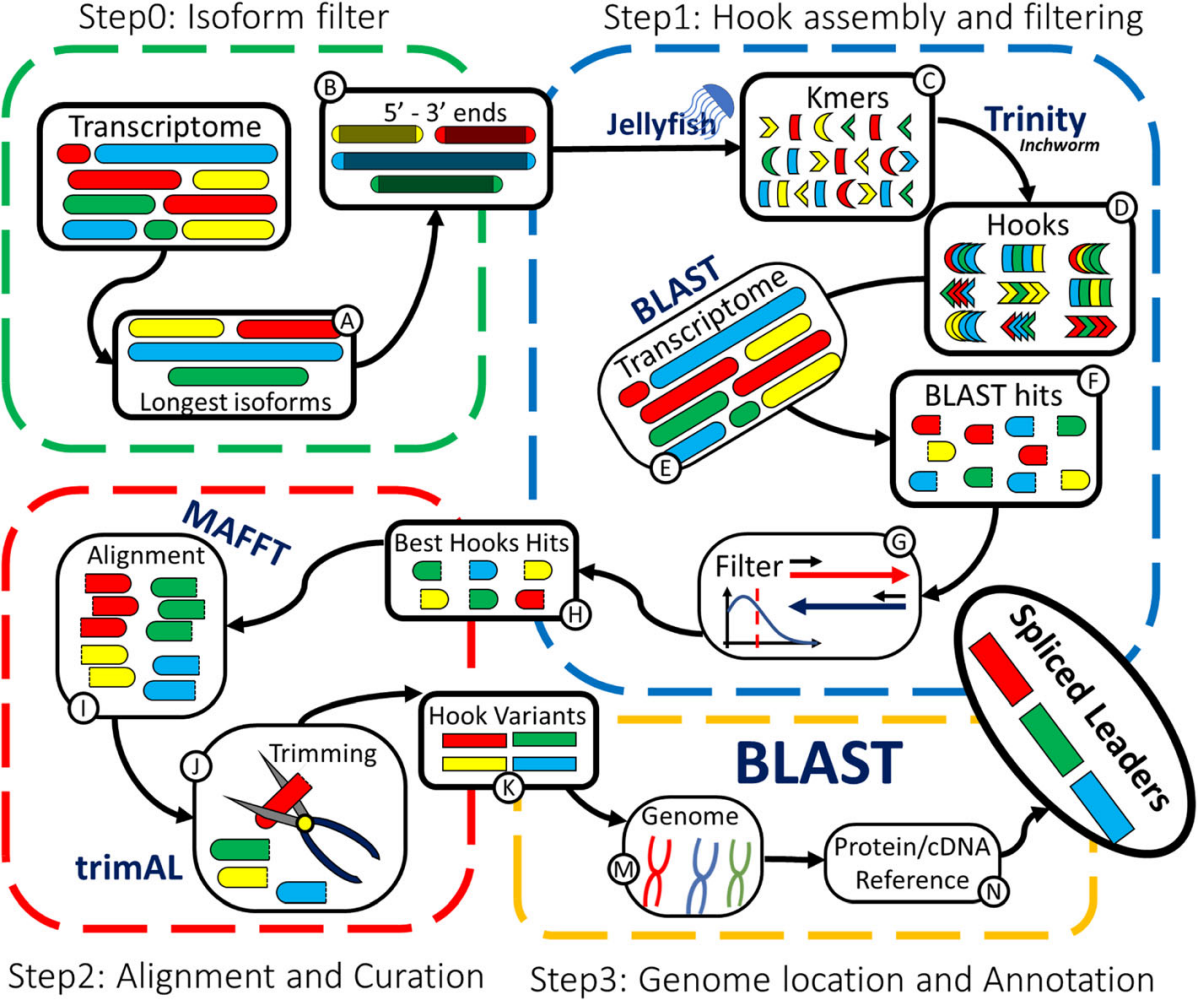
SLFinder workflow. The pipeline is divided into four scripts (Step0 to 3) to facilitate user intervention. Step0 reduces a Trinity assembly redundancy based on the contigs IDs and extract transcripts ends. Step1 generates Hook sequences for the putative Splice Leaders based on the Kmers present on the filtered sequences generated in Step0, identifies matching sequences in the original assembly and selects the Best Hooks based on their overall sequence orientation and frequency. The Best Hook Hits sequences are then aligned and trimmed in Step2 to generate Hook Variants, and Step 3 localizes them on a Reference genome and classifies hits according to the presence of a potential Donor Site. In detail, longer isoforms are retrieved from the transcriptome (A), both terminals regions are keep (B) and kmers are counted with Jellyfish and selected according to their abundance (C). Selected kmers are assembled into Hooks using Inchworm (D) and then Hooks are blasted against the original unfiltered transcriptome (E) and then filtered according to coi and abundance. The Best Hook matching sequences are retrieved (H). These sequences are then aligned with MAFFT (I) and trimmed automatically with trimAL (J), results are Hook Variants that represent possible versions of the repeated sequence that generated the Hooks (including both real polymorphisms and sequencing errors) (K). Step3 locates locus codding for the Hook Variants in the species genome using BLAST and identifies potential donor sites (M). Finally, putative coding sequences are discarded using a third BLAST search against a protein-coding or protein sequences reference databases (N)

The pipeline overview is presented on Fig. 1. First, the redundancy in the de novo assembly transcriptome is reduced in SLFinder-Step0, hereafter referred to as Step0, by retrieving the 5′ and 3′ ends of the longest isoforms of each gene. Isoforms of the same gene are identified based on Trinity’s contigs name convention. Alternative strategies can be implemented (e.g. clustering based on sequence identity) following the manual instructions. Regardless of the chosen method, once redundant sequences are filtered the next step is to identify SLs among the more commonly observed sequences in the transcripts ends. In an ideal situation, the SL sequence should be located at the exact beginning of the assembled transcripts and cleaner results should be obtainable by retrieving from the transcript end a fragment similar in size to the expected length of the SL. However, we noticed that the assemblies used to validate this pipeline often presented non-conserved, mainly low-quality, sequences that preceded the known SL sequence. Instead, Step1 achieves this by counting the kmers present on the filtered sequences (and their reverse complement) with Jellyfish software [47]. Those kmers observed below a given threshold are discarded (by default 0.0005% of the total contigs after filtering, in practice ≈10 contigs, depending on the dataset), and then assembled in longer sequences with Inchworm, Trinity’s first module. The resulting sequences, hereafter referred to as “Hooks”, are a collection of true SL sequences (if present) and every other common sequence found on the filtered transcriptome.

**Fig. 2:**
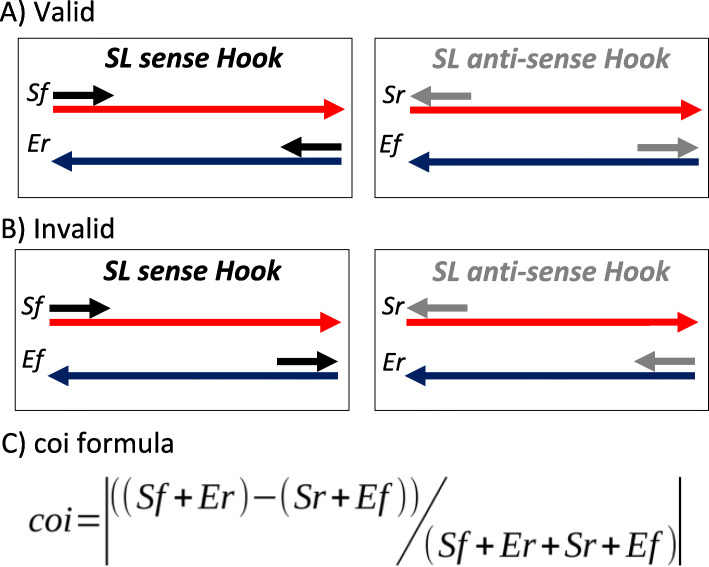
Hook hits orientations and coi filter. Estimation of the concordance orientation index (coi). Hooks potentially representing SLs are classified according to the orientation of their hits on the assembled transcripts with valid and invalid configurations. If SLFinder assumptions are met, the Hook hits should be primarily oriented in one of two valid configurations that depend on the orientation of the assembled transcript (red arrow for sense and blue for anti-sense) and the orientation of the generated Hooks. Note that Hooks could also be assembled in sense (black arrow) or anti-sense (grey arrow). **a** Valid Hook hits orientation and locations. **b** Invalid Hook hits orientation and locations. **c** Coi formula

To further narrow down candidates, Step1 analyzes the location and orientation of matching sequences in the original transcriptome. A Blast search [48] is conducted to “fish out” similar sequences among the transcriptomic contigs (with the “-task blastn-short” option and “-evalue 1e-2”). Hooks are selected according to their number of hits and sequence orientation in the assembled transcriptomes. Since we are working without information on the strand, the transcripts can be assembled either sense or anti-sense; and so, can be the Hooks that are generated from these transcripts. However, if our assumption 3 holds (i.e., the sequence is not a palindrome) and the Hook represents a true SL (meaning that it is located at the 5′ end on the transcript) its hits on the transcriptome should be found in two mutually exclusive configurations, depending on the orientation of the Hook: 1) forward-oriented at the Start of a sense assembled transcript or reverse oriented at the End in an anti-sense one if the Hook was generated in a sense configuration (named Sf and Er orientations, respectively); or 2) reverse oriented at the start of a sense assembled transcript and Forward oriented at the end of an anti-sense one (named Sr and Ef orientations, respectively) (Fig. 2a).

With this in mind, we created a simple consistency orientation index (coi) to evaluate each potential Hook (Fig. 2). A coi equal to 1 means that the Hook’s hits are all oriented in one and only one of the valid configurations. Testing, however, shows Hooks for known SLs can have some hits that do not follow these rules but their coi is always high (i.e., above 0.95; see Results). In addition, tests show that Hooks with few hits on the transcriptome often have a high coi by chance, even when they are not SL sequences. To compensate we also introduce an Observation Count Cutoff (occ) filter that is simply the median of all identified Hooks. Finally, transcripts with multiple hits for the same Hook are excluded and reported separately for user inspection. These transcripts may represent chimeric sequences generated during the assembly process [49, 50] or short repeated sequences. Hooks that pass these filters are selected for further analysis.

The sequences of these selected Hooks, hereafter referred to as Best Hooks, are retrieved for further analysis. The transcript’s ends with a BLAST match (with the “-task blastn-short” option and “-evalue 1e-2”) to each Best Hook are retrieved (from the transcript end until two bases after the match end in order to recover as much sequence from the pSL sequence).

The next filtering step in the pipeline consists of locating and analyzing genomic loci matching the Best Hooks from which they are potentially transcribed (putative SL genes). However, first, it is necessary to address three issues: high redundancy, noisy sequences coming from sequencing and assembly errors, and imprecise pSL delimitation. Without knowing the SL sequence there is no reliable way to address these problems with a one-base precision, nevertheless, Step2 minimizes them by first clustering all sequences according to sequence identity with CD-HIT-EST [51] with a 100% identity threshold by default, followed by alignment with MAFFT [52] using the accuracy oriented method G-INS-I “--globalpair --maxiterate”. Finally, sequences are automatically trimmed with trimAL [53]. The resulting sequences, referred to as “Hook Variants”, represent possible versions of the repeated sequence that generated the Hook (including both real polymorphisms and sequencing errors). Depending on the data, it might be necessary to re-run this step several times with different parameters or even manually curate the sequences before continuing with Step3 (see the software manual for detailed instructions). To facilitate this process, Step2 also generates sequence logos before and after trimming with Weblogos3 [54].

Step3 carries out a BLAST (−task blastn-short) search of the Hook Variants against the provided reference genome to identified pSL coding loci. Since some level of noise is expected in the Hook Variants sequences, even when they represent true SL (see below), the BLAST search is configured with a 100% identity threshold, ungapped, and a high query coverage (90% by default). In practice, these thresholds allow mismatches in the terminal region of the Hook Variant. Once identified, Step3 searches for the existence of a potential donor site and attempts to annotate the region with an external CDS or protein reference with either blastn or tblastx. As a final fail-safe to check the inaccuracy in the pSL delimitation, Step3 takes the following considerations when reporting a potential donor site: 1) It analyses 4 bp surrounding each Hook Variant 3′ end hit in the genome (excluding mismatches in the extremes) looking for a possible splice donor site (“GT”). If one “GT” is found, step3 reports either “5prima” or “3prima” depending on the hit orientation, simplified to “Clear donor site” in this paper. 2) If the longest matching Hook Variant with a donor site overlaps with possible splice donor site (i.e., the sequence ends with a “G” or “GT” that matches with the splice donor site) an “*” is included in the report to indicate that manual inspection is advised. 3) If a potential donor site is found in fewer than 80% of Hook Variants matching a locus, the site is reported as “Unclear”. Finally, a BLAST search between the region surrounding each locus and the provided Protein/cDNA reference dataset is conducted, and loci with matches are discarded. In every step the user can check the Hook Variants and blast results to reconsider or inspect some discarded Hooks.

A putative SL coding Locus was considered valid if a potential donor site was identified and there were no known protein-coding sequences located close to the locus (by default 100pb, this parameter can be changed by the user). Sequences for loci with and without a clear donor site are clustered with CD-HIT-EST in pSL sequences (100% identity Threshold). The final output also includes multiple sequence alignment of each locus done with MAFFT (G-INS-I “--globalpair --maxiterate”) and its original Hook Variants to facilitate manual inspection.

#### Test data

Test data was selected from species according to known presence or absence of SL Trans-splicing, the existence of a reference genome and availability of RNAseq following a poly-A capture protocol. The final species list comprised *Aplysina aerophoba*, *C. elegans* [19], *Ciona intestinalis* [21], *Drosophila melanogaster*, *Hydra vulgaris* [23], *Mus musculus*, *Saccostrea glomerata*, and *Schistosoma mansoni* [18]. *Schistosoma mansoni,* has been reported to have a single SL class with a long sequence (36 bp), represents an ideal scenario to test SLFinder. Meanwhile, *C. intestinalis* with single short SL (16 pb) allows investigating how the pipeline behaves with shorter sequences. Finally, *C. elegans* and *H. vulgaris* have multiple SL sequences (some of them with known sequence diversity among their coding SL-RNAs, e.g. SL2 in *C. elegans*) which will test SLFinder ability to identify and retrieve different SLs when present.

Transcriptomic and genomic data used on these analyses are detailed in Table 1. Non-control samples from experimental studies (i.e. response to pathogens or other stimulus) were discarded. Genomic locus annotation was carried out with the Swiss-Prot database from Uniprot (Downloaded 01/05/2019). In addition, since several genome assemblies are available for *S. mansoni*; mostly based on Protasio et al. 2012 work [55] but improved and annotated following different methodologies for their curation and annotation; SLFinder was tested using 2 reference genomes: one from Wormbase Parasite (WBPS) improved with PacBio data and one from GeneDB that is more fragmented but with several SL-RNA genes annotated.

**Table 1:** Datasets utilized to validate and evaluate SLFinder

Species	Taxon	RNAseq BioProject	Ref. Genome Assembly	Reported SLs
*A. aerophoba*	Porifera	PRJEB26562	GCA_900275595.1^a^	No
*C. elegans*	Nematoda	PRJNA270896	PRJNA13758^b^	Yes
*C. intestinalis*	Urochordata	PRJNA396771	GCF_000224145.3^a^	Yes
*D. melanogaster*	Insecta	PRJNA318586	GCF_000001215.4^a^	No
*H. vulgaris*	Cnidaria	PRJNA497966	Hm105^c^	Yes
*M. musculus*	Vertebrata	PRJNA319673	GCF_000001635.26^a^	No
*S. glomerata*	Molusca	PRJNA487836	GCA_003671525.1	No
*S. masoni*	Plathelminthes	PRJNA225599	PRJEA36577^b^ and GeneDB	Yes

^a^ Available on NCBI database

^b^ Available on WBPS database

^c^ Hydra 2.0 Genome Project

Read quality for RNAseq data was assessed with FastQC [56] and low quality bases along with adapter sequences were removed with Trimmomatic v0.36 [57] (options: ILLUMINACLIP: TruSeq3-PE.fa:2:30:10, SLIDINGWINDOW: 5:20 and MINLEN: 50). Transcriptomes were de novo assembled with Trinity v2.8.3, without read normalization.

#### Bioinformatic analysis and pipeline evaluation

Analyses were carried out in a desktop computer with 96Gb of RAM and 32 threads/16 cores (only 4 threads were used on each run). Program versions used are listed on Table 2 with default parameters (with exception of *C. intestinalis*). Pipeline accuracy was tested by sequence comparisons with known SL sequences (Additional file 1), verifying the match of the predicted SL locus with the annotated SL within 100 bp range. This comparison was done using gffread [58]. In addition, each potential locus was manually inspected, and “seqkit locate” was utilized to verify the transcripts carrying specific pSL sequences in order to detect and categorize artefacts. Figures of sequence alignments were generated with BioEdit v7.0.5.3 [59].

**Table 2:** List of programs and software packages utilized by SLFinder, including the version utilized in this paper and the basic tasks they carry out

Program	Version	Tasks
Blast	v2.6.0	Sequence searches against Transcriptome assemblies, Genome and Protein reference database.
cd-hit-est	v4.7	Sequence clustering to simplify results and reduce runtimes
Jellifish	v2.2.6	Kmer counts
MAFFT	v7.307	Sequence Alignment
Seqkit	v0.10.0	Basic sequence manipulation
trimAl	v1.2rev59	Hook Variant generation by automatic trimming
Trinity	v2.8.3	Hook assembly from Kmers
Weblogos	v3.6.0	Sequence Logos generation to facilitate manual curation

## Results

A total of 32 transcriptomes (9 from *A. aerophoba*, 6 from *C. elegans*, 3 from *C. intestinalis*, 4 from *D. melanogaster*, 5 from *H. vulgaris*, 2 from *M. musculus*, 1 from *S. glomerate*, and 2 *S. mansoni*) were assembled and analyzed (Basic descriptor metrics are shown in Additional file 2). Running times per step where highly dependent on the dataset (Table 3) mainly depending on the number of reads to process. No Hook sequence passed the coi filter in Step1 for the species without known SLs *A. aerophoba, D. melanogaster*, *M. musculus* and *S. glomerata* (Additional file 3).

**Table 3:** SLFinder steps performance for all datasets

Data Set	Step0	Step1	Step2	Step3	Total
*A. aerophoba*	32 m 12 s	0 m 34 s	X	X	32 m 46 s
*C. elegans*	4 m 23 s	0 m 24 s	13 m 25 s	1 m 08 s	19 m 46 s
*C. intestinalis*	4 m 29 s	1 m 54 s	0 m 03 s	0 m 43 s	7 m 09 s
*D. melanogaster*	5 m 36 s	0 m 20s	X	X	5 m 56 s
*H. vulgaris*	15 m 17 s	57 m 26 s	19 m 58 s	14 m 35 s	1 h 47 m 16 s
*M. musculus*	7 m 14 s	2 m 10s	X	X	7 m 24 s
*S. glomerata*	24 m 24 s	0 m 25 s	X	X	24 m 59 s
*S. mansoni*	6 m 53 s	0 m 38 s	0 m 06 s	3 m 03 s	10 m 40s

Positive results were identified for the species *C. elegans*, *Hydra vulgaris* and *S. mansoni*, all with previously described SL. SLFinder also identified the SL reported for *C. intestinalis* after changing the parameters to account for short SLs (15-base kmer length, 14 Inchworm assembly kmer, and no filtering according to the median count value).

In the following sections we will describe the results obtained by each Step of SLFinder (Step 1 Hook generation and filtering, Step 2 Hook Variant trimming, and Step 3 putative SL (pSL) loci identification) on each positive dataset. Since the intent of this software is novel SL identification, we will focus on features of SLFinder reports that depart from the true SL sequence (e.g. longer/shorter sequences than expected and potential false positives results).

### *Caenorhabditis elegans* dataset

**Fig. 3:**
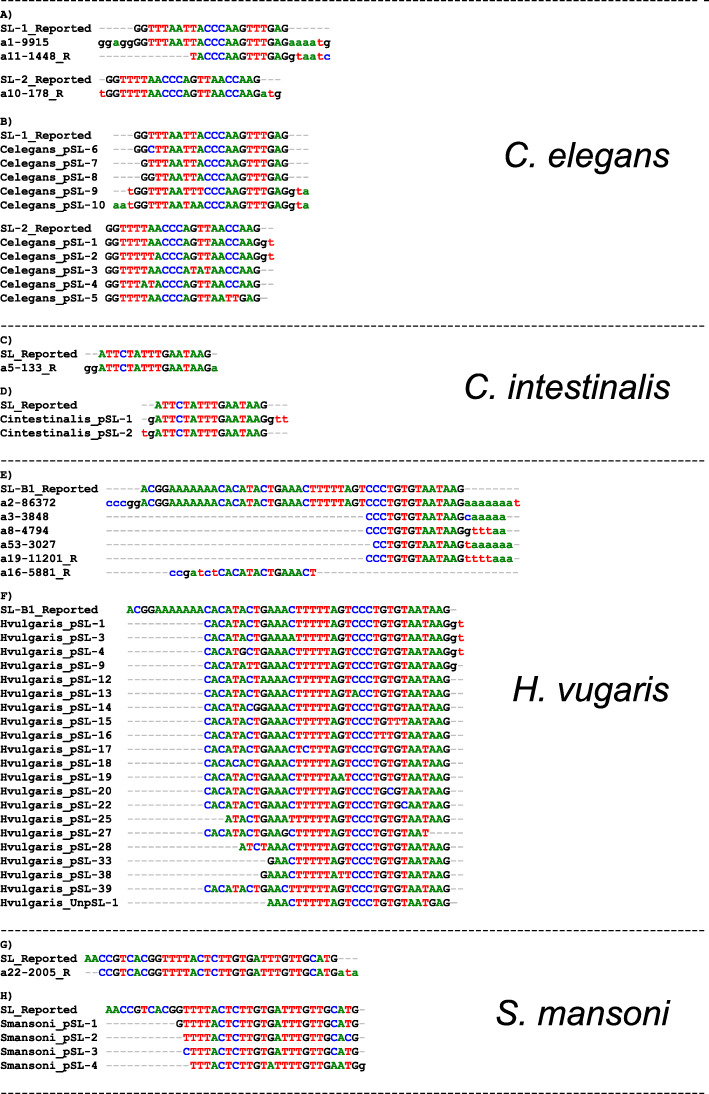
SLFinder predictions for all datasets. SLFinder summary results and known SL sequences reported in analyzed species. For each species, the figure shows selected Best Hooks found in Step 1 (**a**, **c**, **e**, and **g**), and predicted putative SLs (pSL) after Steps 2 and 3 (**b**, **d**, **f**, and **h**). “_R” referred to reverse complement sequence of the Best Hook found. Figures of sequence alignments were generated with BioEdit v7.0.5.3 [59]

A total of 13 hooks were generated in Step1, three of which passed both the coi and the occ filters and resulted in 246 different Hook Variants after Step2. Comparison with known SL sequences showed that the Best Hooks “a1–9915” and “a11–1448” corresponded to the known SL-1, while “a10–178” to SL-2 (Fig. 3a).

Step3 identified 26 putative pSL loci in the reference genome, 18 of which were previously reported as SL-RNA genes in the genome annotation (Additional file 4). Thirteen pSL were reported as having a clear potential donor site and were later clustered into 8 sequences; hereafter referred to as Celegans_pSL-(1 to 8). Another 10 loci were reported as Unclear due to the presence of several bases in the 3′ region in several variants for the Hook “a1–9915” that overlapped with the splice donor site (Additional file 5). Most of these loci were located on Chromosome V in a cluster of ≈13 kb and were grouped into a single sequence identical to Celegans_pSL-7. In addition, Locus-5 and -26 were reported without a donor site and Locus-25 was not analyzed because SLFinder failed to determine its orientation due to low count numbers in the transcriptome. Manual inspection showed that both Locus-25 and Locus-26 have a potential donor site masked by a three base extension in the 3′ end (GTA) of the only matching Hook Variant for each, hereafter referred to as Celegans_pSL-9 and -10, respectively (see similar cases in Additional file 5).

Potential Spliced Leaders Celegans_pSL-6, − 7, − 8, − 9, and − 10 match the previously described SL-1 and their nucleotide differences were limited to the 5′ region, whereas Celegans_pSL-1, − 2, − 3, − 4 and − 5 represent different variants of SL-2 and are slightly more diverse in their nucleotide sequences (Fig. 3b). These observations are in concordance with the genome annotation and previous results for *C. elegans* [2].

Site-specific observations of these loci are included in Additional file 4. Of particular relevancy are Locus-12 and -20, both display partial repetitions of SL-1 following the reported hit (Additional file 6). Verification of the functionality of these SLs loci is beyond the scope of this paper and the capabilities of SLFinder, a pattern search against the reads only identified six read pairs bearing Locus-12 repeat across all samples.

In Summary, SLFinder identified both SL classes, SL-1 and SL-2, previously reported for *C. elegans* and located several of their described SL-RNAs loci (10 for SL-1 and 8 for SL-2), in addition to five not previously annotated copies of SL. While verifying the functionality of these new SL-RNAs is beyond the scope of this paper, our results suggest that at least two of them (Locus-12 and -20) are presumed to be pseudogenes due to the presence of fragments from SL-1 following the 3′ end.

### *Ciona intestinalis* dataset

Using the modified parameters (15-base kmer length, 14-base Inchworm assembly kmer and removing occ filtering), Step1 generated 81 Hooks but only “a5–133” passed the coi filter. Twenty-three Hook Variants were identified for “a5–133” in Step2. Results show that the Hook matches the sequence of the previously reported SL [21] (Fig. 3c).

Step3 identified 38 putative loci in the genome, 23 of which have a predicted protein-coding gene in the matched region according to the available annotation (Additional file 7). Long non-coding RNA (lncRNA) are annotated surrounding Locus-8, − 12, and − 15 in this dataset (XR_717275.3, XR_003396022.1 and XR_003396339.1 respectively), but their functions are unknown and only Locus-12 is encompassed by the lncRNA included by its hit. Fourteen pSL were reported with a clear potential donor site and were grouped into two clusters; hereafter named as Cintestinalis_pSL-1 and -2; that differ on their extension toward both sequence ends (Fig. 3d). The former shows a 3′ extension “GTT” which overextends the expected donor site for the SL and ends next to another “GT” in the genome. Detailed observations are included in Additional file 7.

Despite its shorter size, once the software parameters were properly fine-tuned, SLFinder was able to recover the reported SL sequence for this species. However, the results are not as clear as other datasets analyzed, indicating that these conditions are near the limits of what is possible to obtain with this strategy.

### Hydra vulgaris dataset

For this species Step1 generated 31 Hooks, 6 of which passed both coi and frequency filters and their matches in the transcriptome were processed on 385 Hook Variants. Comparison with known SL sequences for the species shows that the longest Hook “a2–86,372” matches SL-B1 (reported in [36]); while hooks “a3–3848”, “a8–4794”, “a16–5881”, “a19–11,201” and “a53–3028” match only the terminal region (Fig. 3e). Unfortunately, the 5′ region of the observed Hook Variants, from 16 to 32 bases, was lost in Step2 during trimming (Additional file 5d).

Step3 identified 239 loci in the genome many of which were found in close proximity to annotated protein-coding regions (Additional file 8). 93 loci were reported with a clear donor site (“Clear”) and 59 with an unclear donor site (“Unclear”). The former was clustered in 37 pSL sequences, Hvulgaris_pSL-(1 to 37), and the latter in 10, Hvulgaris_UnpSL-(1 to 10). Hvulgaris_UnpSL-4 has an identical sequence to Hvulgaris_pSL-1, Hvulgaris_UnpSL-3 to Hvulgaris_pSL-6, and Hvulgaris_UnpSL-7 to Hvulgaris_pSL-9 (Additional file 9). In addition, 5 loci were not analyzed because SLFinder failed to determinate their orientation due to low counts in the transcriptome. Among these, Locus-46 and -112 display a potential donor site and are included in the further discussion as Hvulgaris_pSL-38 and -39 respectively.

The manual inspection revealed several issues that suggest they are most likely non-functional versions of SLs that guarantee further analysis. For the purposes of presenting the tool, however, they were considered non-functional. Removing them reduces the pSL unique sequences to 21 (Fig. 3f) (see the full set of pSLs generated by SLFinder in Additional file 9 and detailed observations in Additional file 8). Note that many pSL loci displayed a donor site that overlaps with the known last base of the SL (as previously described for *C. elegans*), while others presented extensions that led to an alternative “GT” (as previously described for *C. intestinalis*) without including the expected donor site. While is possible that the latter pSLs represent longer than already reported SL sequences, testing this will require additional studies that are beyond the scope of this paper. Furthermore, an inspection of the transcripts bearing these sequences indicates that these pSL loci match some transcripts for several bp after the pSL sequence (Data not shown) raising further doubts on their functionality. Lastly, 28 loci showed partial repeats of SL sequences, including some of the previously reported SLs (SL-D, −F, and -G) that were not recovered by SLFinder (Additional file 6).

In summary, *H. vulgaris* was the most complex dataset analyzed, with several potential pseudogenes for the SL-RNAs identified. This is, likely in no small extent, related to their complex evolutionary history [36]. Unfortunately, SLFinder failed to identify the other 6 SL reported for the species [36]. A pattern search with seqkit locate of the terminal region of these SLs on the original fastq files indicates a marginal presence of SL-B2, SL-B3, SL-B4, SL-D and SL-G in the dataset, so the most probable cause of this false negatives is their low prevalence in the analyzed RNAseq data (Data not shown).

### *Schistosoma mansoni* dataset

One Hook (“a22–2005”) out of 30 generated in Step1 passed both coi and frequency filters and was then processed into 11 Hook Variants by Step2. Comparison with the known SL sequence for *S. mansoni* shows that this Hook represents the reverse complement of the described SL in almost its entirety (Fig. 3g). As with the *H. vulgaris* dataset, part of the 5′ region of the Hook that was recovered was lost during trimming in Step2 due to the poor alignment quality of this region. This could be at least partially explained by missing information and high variability among the retrieved sequences in the transcriptome assemblies (Data not shown).

When using the WBPS reference genome, Step3 identified 132 pSL loci, only 13 in the proximity to protein-coding genes (Additional file 10). Most of them showed a clear donor site and were clustered in 3 groups; hereafter referred to as Smansoni_pSL-(1 to 3). The remaining 9 loci were reported as lacking a potential donor site. This was confirmed by manual inspection in all cases except for Locus-128, in which the donor site was masked by the retention of 3 bp on the 3′ end of the generated Hook Variant; hereafter referred to as Smansoni_pSL-4. All four pSL are shown in Fig. 3h while loci coordinates and observations are reported in Additional file 10. Note that only Smansoni_pSL-1 was encoded by several loci. On the other hand, Smansoni_pSL-2 had a substitution in the terminal ATG of the SL. This ATG was reported as completely conserved in all studied Platyhelminthes (see [5]). A pattern search of the terminal region of this pSL reveals a marginal presence on the reads from both sequenced samples, indicating very low expression of this SL variant in the dataset (Data not shown).

Surprisingly, only 22 pSL loci were identified when using the GeneDB reference genome (Additional file 11). Fifteen of these presented a clear potential donor site and were clustered in the same three pSL classes found using the WBPS reference genome (see above), including Smansoni_pSL-4 (Data not shown). Five pSL coding loci were already reported as SL-RNA coding genes, including one locus that was reported without a donor site because of missing information in the reference genome.

In the case of *S. mansoni*, SLFinder identified the known SLs, including one possible pseudogene, with the only drawback of a partial recovery of its 5′ region. Results also show the importance of the Reference Genome, as illustrated by the number of pSL loci found in the assemblies of WBPS and GeneDB.

## Discussion

### Considerations when using SLFinder

The strategy presented here, although effective, has shortcomings that originate from the input data and the minimal assumptions regarding the SL sequences. SLFinder requires enough SL exon sequences to be present in the de novo transcriptome assembly. This may be an important issue when considering the widely distributed poly-A enrichment strategy for RNAseq in eukaryotes, nevertheless, our results clearly show that identifying SL sequences and loci is possible in real datasets. Short SL sequences, poor data quality, and the inappropriate reference genome, or a combination of the three may also be issues to consider. See for instance the results of *C. intestinalis* dataset, which could be handled however with specific parameters settings. When dealing with such cases we recommend changing kmer size, ideally using similar organisms a guideline, and annotate every hit for a Hook with a high coi value. Bear in mind that because of these limitations, negative results should not be considered evidence of absence of SL Trans-Splicing.

In addition, the lack of reliance on known SL sequences combined with the approach taken to generate Hook Variants are the source of the issues in identifying the donor site described in Results. Basically, the problem is how to answer the question: “Where does the sequence end when the sequence is unknown?”. SLFinder solves this issue by trimming according to alignment quality and then localizing them in the reference genome for further pinpointing the SL extension. While most of the issues with automatic trimming (see [60]) don’t apply in this context, a side effect of this strategy is the addition of non-SL bp if they are present in enough transcripts, along with a common loss of the SL 5′ region during the trimming of Hook hits (both observed in the Results section). Nevertheless, both drawbacks can be properly addressed with an informed user intervention that is facilitated by SLFinder modularity, either by adjusting trimAL parameters or manually processing the alignments (Note that these modifications might affect Step3 results as some divergent pSL Loci will be lost).

The quality of the reference genome and other biological features of the species play an important role in SLFinder accuracy and performance. As stated before, the reference genome is a key piece of information when pinpointing the pSL sequence and filtering out Hook Variants generated due to sequencing and/or assembly errors. This is clearly shown in the analyses of *H. vulgaris* and *S. mansoni* datasets. On the one hand, SL prediction in *H. vulgaris* was far from straightforward given the high abundance of pSL coding loci found, many of which are likely false positives. This result may be explained, at least in part, by the high prevalence of transposable elements in their genome [36]. In the case of *S. mansoni* the differences observed between WBPS and GeneDB genome assemblies may explain the different results obtained with SLFinder for this species. A better assembly may help identify more SL loci, as is the case of WBPS assembly. Note that PacBio technology was used to improve assembly quality in this assembly [61].

In the absence of a reference genome, the Hook sequences generated during Step1 and the Hook Variants in Step2 offer a good alternative, but it would require validation based on homology (SL sequences from other closely related taxa) or wet lab experimental approaches.

### Advantages of SLFinder

Taxon sampling bias has been a constant issue in the study of SL trans-splicing across the tree of life. For example, Bitar et al. study conducted a study based on BLAST searches against public databases and identified mostly SL-1 like sequences in the phylum Nematoda. Results included species like *Globodera rostochiensis* that possess known divergent SL sequences [38] and *Heterodera glycines* for which more SL classes were latter described [31]. SLFinder represents a solution to this problem by providing a straightforward method to identify pSL sequences that is not based on sequence homology.

The use of over-represented kmers to identify regulatory regions is not a new approach for exploratory analysis of DNA sequences [62] and was applied to identify SL sequences before [36]. However, the novel but simple filters implemented in SLFinder allowed the easy recovery of known SL exonic sequences of the four species with this splicing mechanism in just a few hours; and in the case of *C. elegans* and *S. mansoni* even identifying the known SL-RNA coding loci. Only the *C. intestinalis* dataset required a fine-tuning of SLFinder parameters to account for a shorter than expected SL sequences.

Potential SLs sequences identified with this pipeline can be validated through experimental procedures like RT-PCR or 5′ RACE (e.g. [31, 37]) or can be used as input data for other informatics analyses like the ones implemented by SLQuant [34] and, UTRme [33]. Even a simple pattern search (e.g. [31]) could be used to identify the acceptor genes in order to further analyze mRNA maturation in the species of interest. The identified putative SLs coding loci can be used to further validate the SL-RNA by looking for the sm site or the RNA secondary structure [1, 5, 37].

## Conclusion

SLFinder offers a practical alternative for the discovery of novel SL sequences aside from homology searches or fortuitous identification. This modular pipeline was proved with freely available RNAseq data for organisms with and without reported Splice Leader sequences with very good results. Putative SLs found by SLFinder can be later refined regarding their exact length and confirmed through additional bioinformatics analyses and wet lab experiments. This software represents a step forward toward a more comprehensive understanding of the distribution of SL Trans-Splicing in the tree of life, its evolutionary history and importance.

## Supplementary information

**Additional file 1: Supplementary Table 1.** Reported SL sequences for the species *C. elegans*, *C. intestinalis*, *H. vulgaris* and *S. mansoni*.

**Additional file 2: Supplementary Table 2.** Basic descriptors of the analyzed datasets. Data Set, GenBank SRA ID, N° Reads, N° Trimmed Reads, Assembled Bases, Total Transcripts, N50, Median Contig Length, Average Contig Length, Genes predicted by Trinity (Trinity Genes) and GC content.

**Additional file 3: Supplementary Table 3.** Hook hits obtained for each dataset (Step 1 - F in Fig. 1). Hit counts according to position and orientation in the contig, coi score and observation count cutoff (occ) are indicated. Selected Best Hooks are highlighted in bold. Results for *C. intestinalis* include those obtained with default and custom values (see main text).

**Additional file 4: Supplementary Table 4.** Putative SLs loci identified by SLFinder in the *C. elegans* dataset. Previously reported SL-RNAs are also indicated.

**Additional file 5: Supplementary Figure 1.** Common issues to consider when utilizing SLFinder.

**Additional file 6: Supplementary Figure 2.** Non-functional SL loci found during SLFinder analyses.

**Additional file 7: Supplementary Table 5.** Putative SL loci identified by SLFinder in the *C. intestinalis* dataset. Annotated lncRNA and protein-coding genes closer than 100 pb are also indicated.

**Additional file 8: Supplementary Table 6.** Putative SL loci identified by SLFinder in the *H. vulgaris* dataset. Annotated protein-coding genes closer than 100 pb are also indicated.

**Additional file 9: Supplementary Figure 3.** Sequences of putative SL identified in *H. vulgaris* dataset.

**Additional file 10: Supplementary Table 7.** Putative SL loci identified by SLFinder in the *S. mansoni* dataset using Wormbase’s genome assembly as reference (WBPS). Annotated protein-coding genes closer than 100 bp are indicated.

**Additional file 11: Supplementary Table 8.** Putative SL loci identified by SLFinder in the *S. mansoni* dataset using GeneDB’s genome assembly as reference. Annotated protein-coding genes closer than 100 bp are indicated.

## Abbreviations

coi: Consistency orientation index
Ef: Hook match at the End of the transcript in Forward orientation
Er: Hook match at the End of the transcript in Reverse orientation
NGS: Next Generation Sequencing
occ: Observation Count Cutoff
pSL: Putative Splice Leader
Sf: Hook match at the Start of the transcript in Forward orientation
SL: Spliced Leader
Sr: Hook match at the Start of the transcript in Reverse orientation
WBPS: Wormbase Parasite
