# Supplementary material for: SLFinder, a pipeline for the novel identification of splice-leader sequences: a good enough solution for a complex problem
Source: BMC Bioinformatics. 2020 Jul 8;21:293. doi: 10.1186/s12859-020-03610-6 (PMC7346339; doi:10.1186/s12859-020-03610-6)
Supplement: Supplementary file 5 — Additional file 5: Supplementary Figure 1. Common issues to consider when utilizing SLFinder. [file 12859_2020_3610_MOESM5_ESM.pptx]

## Slide 1
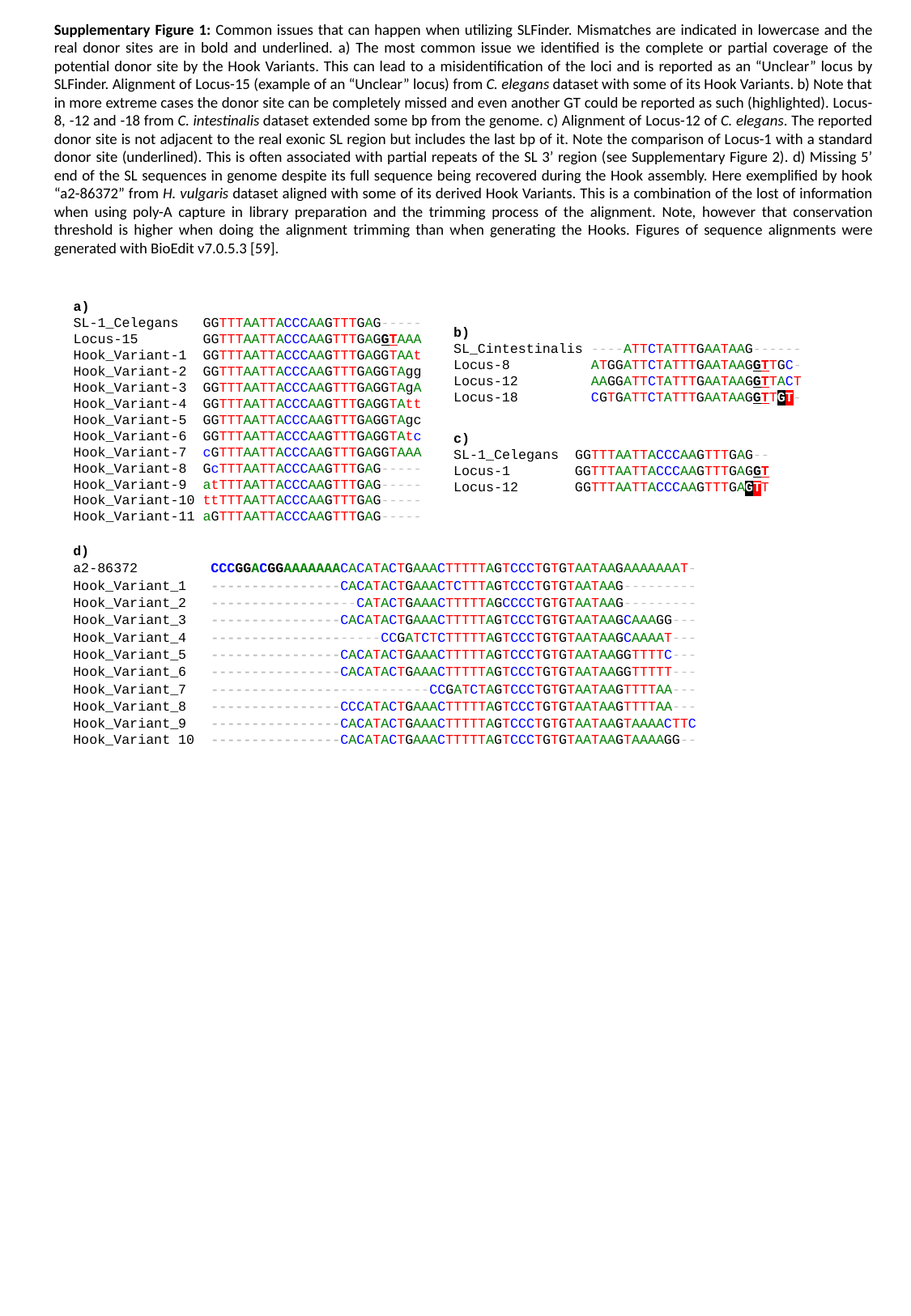

Supplementary Figure 1: Common issues that can happen when utilizing SLFinder. Mismatches are indicated in lowercase and the real donor sites are in bold and underlined. a) The most common issue we identified is the complete or partial coverage of the potential donor site by the Hook Variants. This can lead to a misidentification of the loci and is reported as an “Unclear” locus by SLFinder. Alignment of Locus-15 (example of an “Unclear” locus) from C. elegans dataset with some of its Hook Variants. b) Note that in more extreme cases the donor site can be completely missed and even another GT could be reported as such (highlighted). Locus-8, -12 and -18 from C. intestinalis dataset extended some bp from the genome. c) Alignment of Locus-12 of C. elegans. The reported donor site is not adjacent to the real exonic SL region but includes the last bp of it. Note the comparison of Locus-1 with a standard donor site (underlined). This is often associated with partial repeats of the SL 3’ region (see Supplementary Figure 2). d) Missing 5’ end of the SL sequences in genome despite its full sequence being recovered during the Hook assembly. Here exemplified by hook “a2-86372” from H. vulgaris dataset aligned with some of its derived Hook Variants. This is a combination of the lost of information when using poly-A capture in library preparation and the trimming process of the alignment. Note, however that conservation threshold is higher when doing the alignment trimming than when generating the Hooks. Figures of sequence alignments were generated with BioEdit v7.0.5.3 [59].
a)
SL-1_Celegans GGTTTAATTACCCAAGTTTGAG-----
Locus-15 GGTTTAATTACCCAAGTTTGAGGTAAA
Hook_Variant-1 GGTTTAATTACCCAAGTTTGAGGTAAt
Hook_Variant-2 GGTTTAATTACCCAAGTTTGAGGTAgg
Hook_Variant-3 GGTTTAATTACCCAAGTTTGAGGTAgA
Hook_Variant-4 GGTTTAATTACCCAAGTTTGAGGTAtt
Hook_Variant-5 GGTTTAATTACCCAAGTTTGAGGTAgc
Hook_Variant-6 GGTTTAATTACCCAAGTTTGAGGTAtc
Hook_Variant-7 cGTTTAATTACCCAAGTTTGAGGTAAA
Hook_Variant-8 GcTTTAATTACCCAAGTTTGAG-----
Hook_Variant-9 atTTTAATTACCCAAGTTTGAG-----
Hook_Variant-10 ttTTTAATTACCCAAGTTTGAG-----
Hook_Variant-11 aGTTTAATTACCCAAGTTTGAG-----
b)
SL_Cintestinalis ----ATTCTATTTGAATAAG------
Locus-8 ATGGATTCTATTTGAATAAGGTTGC-
Locus-12 AAGGATTCTATTTGAATAAGGTTACT
Locus-18 CGTGATTCTATTTGAATAAGGTTGT-
c)
SL-1_Celegans GGTTTAATTACCCAAGTTTGAG--
Locus-1 GGTTTAATTACCCAAGTTTGAGGT
Locus-12 GGTTTAATTACCCAAGTTTGAGTT
d)
a2-86372 CCCGGACGGAAAAAAACACATACTGAAACTTTTTAGTCCCTGTGTAATAAGAAAAAAAT-
Hook_Variant_1 ----------------CACATACTGAAACTCTTTAGTCCCTGTGTAATAAG---------
Hook_Variant_2 ------------------CATACTGAAACTTTTTAGCCCCTGTGTAATAAG---------
Hook_Variant_3 ----------------CACATACTGAAACTTTTTAGTCCCTGTGTAATAAGCAAAGG---
Hook_Variant_4 ---------------------CCGATCTCTTTTTAGTCCCTGTGTAATAAGCAAAAT---
Hook_Variant_5 ----------------CACATACTGAAACTTTTTAGTCCCTGTGTAATAAGGTTTTC---
Hook_Variant_6 ----------------CACATACTGAAACTTTTTAGTCCCTGTGTAATAAGGTTTTT---
Hook_Variant_7 ---------------------------CCGATCTAGTCCCTGTGTAATAAGTTTTAA---
Hook_Variant_8 ----------------CCCATACTGAAACTTTTTAGTCCCTGTGTAATAAGTTTTAA---
Hook_Variant_9 ----------------CACATACTGAAACTTTTTAGTCCCTGTGTAATAAGTAAAACTTC
Hook_Variant 10 ----------------CACATACTGAAACTTTTTAGTCCCTGTGTAATAAGTAAAAGG--
