# Supplementary material for: SLFinder, a pipeline for the novel identification of splice-leader sequences: a good enough solution for a complex problem
Source: BMC Bioinformatics. 2020 Jul 8;21:293. doi: 10.1186/s12859-020-03610-6 (PMC7346339; doi:10.1186/s12859-020-03610-6)
Supplement: Supplementary file 6 — Additional file 6: Supplementary Figure 2. Non-functional SL loci found during SLFinder analyses. [file 12859_2020_3610_MOESM6_ESM.pptx]

## Slide 1
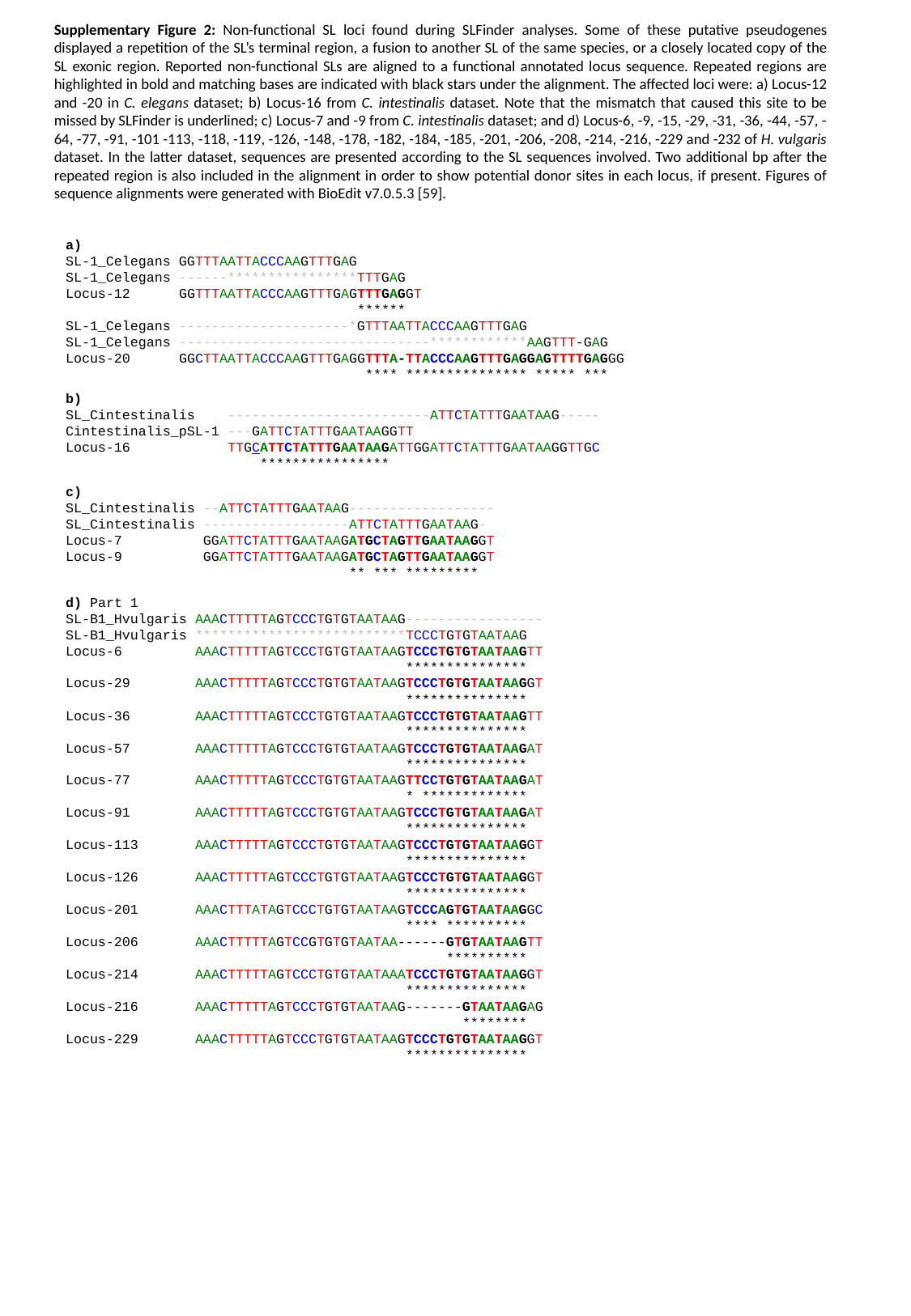

Supplementary Figure 2: Non-functional SL loci found during SLFinder analyses. Some of these putative pseudogenes displayed a repetition of the SL’s terminal region, a fusion to another SL of the same species, or a closely located copy of the SL exonic region. Reported non-functional SLs are aligned to a functional annotated locus sequence. Repeated regions are highlighted in bold and matching bases are indicated with black stars under the alignment. The affected loci were: a) Locus-12 and -20 in C. elegans dataset; b) Locus-16 from C. intestinalis dataset. Note that the mismatch that caused this site to be missed by SLFinder is underlined; c) Locus-7 and -9 from C. intestinalis dataset; and d) Locus-6, -9, -15, -29, -31, -36, -44, -57, -64, -77, -91, -101 -113, -118, -119, -126, -148, -178, -182, -184, -185, -201, -206, -208, -214, -216, -229 and -232 of H. vulgaris dataset. In the latter dataset, sequences are presented according to the SL sequences involved. Two additional bp after the repeated region is also included in the alignment in order to show potential donor sites in each locus, if present. Figures of sequence alignments were generated with BioEdit v7.0.5.3 [59].
a)
SL-1_Celegans GGTTTAATTACCCAAGTTTGAG
SL-1_Celegans ------****************TTTGAG
Locus-12 GGTTTAATTACCCAAGTTTGAGTTTGAGGT
 ******
SL-1_Celegans ---------------------*GTTTAATTACCCAAGTTTGAG
SL-1_Celegans -------------------------------************AAGTTT-GAG
Locus-20 GGCTTAATTACCCAAGTTTGAGGTTTA-TTACCCAAGTTTGAGGAGTTTTGAGGG
 **** *************** ***** ***
b)
SL_Cintestinalis -------------------------ATTCTATTTGAATAAG-----
Cintestinalis_pSL-1 ---GATTCTATTTGAATAAGGTT
Locus-16 TTGCATTCTATTTGAATAAGATTGGATTCTATTTGAATAAGGTTGC
 ****************
c)
SL_Cintestinalis --ATTCTATTTGAATAAG------------------
SL_Cintestinalis ------------------ATTCTATTTGAATAAG-
Locus-7 GGATTCTATTTGAATAAGATGCTAGTTGAATAAGGT
Locus-9 GGATTCTATTTGAATAAGATGCTAGTTGAATAAGGT
 ** *** *********
d) Part 1
SL-B1_Hvulgaris AAACTTTTTAGTCCCTGTGTAATAAG-----------------
SL-B1_Hvulgaris **************************TCCCTGTGTAATAAG
Locus-6 AAACTTTTTAGTCCCTGTGTAATAAGTCCCTGTGTAATAAGTT
 ***************
Locus-29 AAACTTTTTAGTCCCTGTGTAATAAGTCCCTGTGTAATAAGGT
 ***************
Locus-36 AAACTTTTTAGTCCCTGTGTAATAAGTCCCTGTGTAATAAGTT
 ***************
Locus-57 AAACTTTTTAGTCCCTGTGTAATAAGTCCCTGTGTAATAAGAT
 ***************
Locus-77 AAACTTTTTAGTCCCTGTGTAATAAGTTCCTGTGTAATAAGAT
 * *************
Locus-91 AAACTTTTTAGTCCCTGTGTAATAAGTCCCTGTGTAATAAGAT
 ***************
Locus-113 AAACTTTTTAGTCCCTGTGTAATAAGTCCCTGTGTAATAAGGT
 ***************
Locus-126 AAACTTTTTAGTCCCTGTGTAATAAGTCCCTGTGTAATAAGGT
 ***************
Locus-201 AAACTTTATAGTCCCTGTGTAATAAGTCCCAGTGTAATAAGGC
 **** **********
Locus-206 AAACTTTTTAGTCCGTGTGTAATAA------GTGTAATAAGTT
 **********
Locus-214 AAACTTTTTAGTCCCTGTGTAATAAATCCCTGTGTAATAAGGT
 ***************
Locus-216 AAACTTTTTAGTCCCTGTGTAATAAG-------GTAATAAGAG
 ********
Locus-229 AAACTTTTTAGTCCCTGTGTAATAAGTCCCTGTGTAATAAGGT
 ***************

## Slide 2
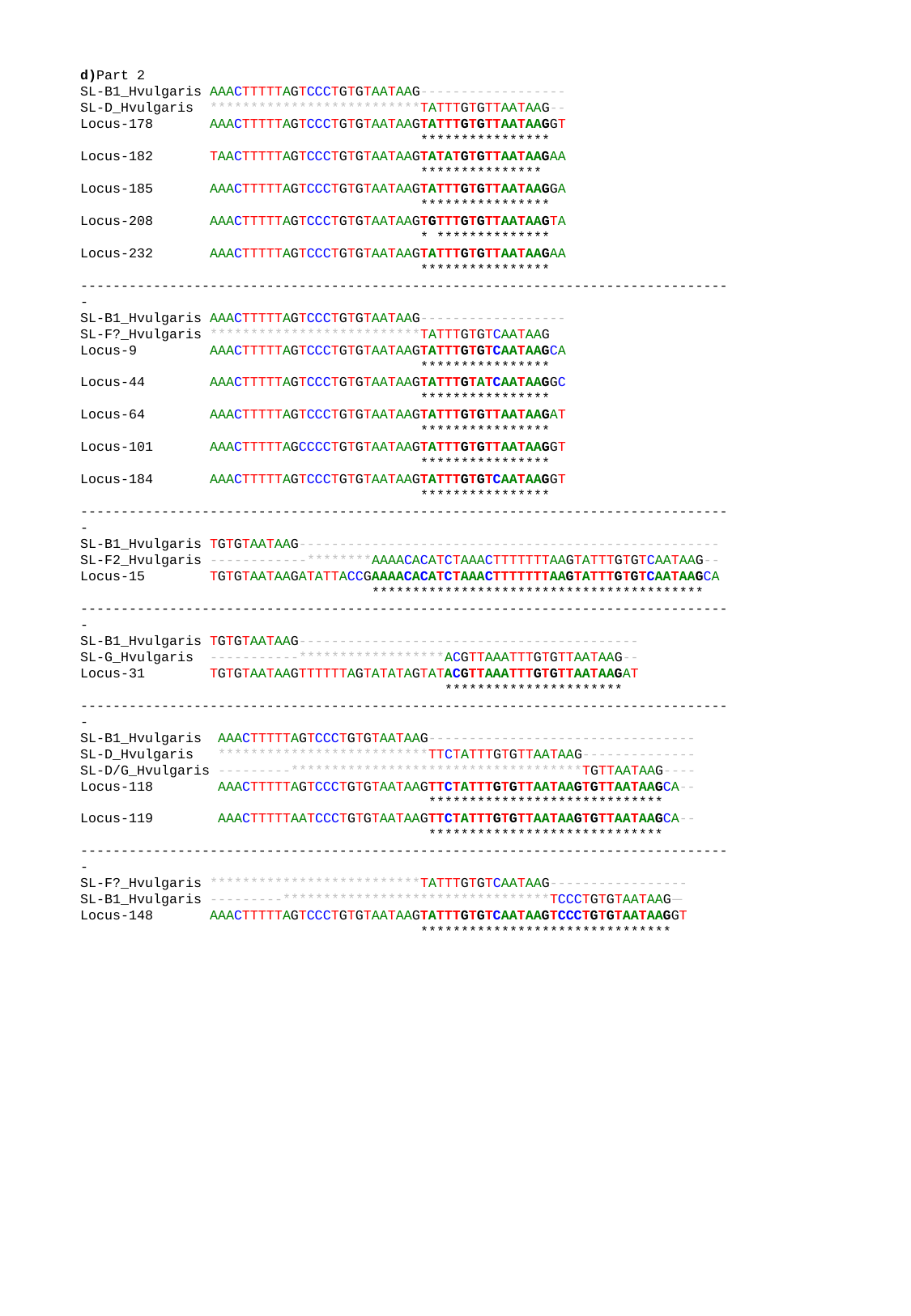

d)Part 2
SL-B1_Hvulgaris AAACTTTTTAGTCCCTGTGTAATAAG------------------
SL-D_Hvulgaris **************************TATTTGTGTTAATAAG--
Locus-178 AAACTTTTTAGTCCCTGTGTAATAAGTATTTGTGTTAATAAGGT
 ****************
Locus-182 TAACTTTTTAGTCCCTGTGTAATAAGTATATGTGTTAATAAGAA
 ***************
Locus-185 AAACTTTTTAGTCCCTGTGTAATAAGTATTTGTGTTAATAAGGA
 ****************
Locus-208 AAACTTTTTAGTCCCTGTGTAATAAGTGTTTGTGTTAATAAGTA
 * **************
Locus-232 AAACTTTTTAGTCCCTGTGTAATAAGTATTTGTGTTAATAAGAA
 ****************
---------------------------------------------------------------------------------
SL-B1_Hvulgaris AAACTTTTTAGTCCCTGTGTAATAAG------------------
SL-F?_Hvulgaris **************************TATTTGTGTCAATAAG
Locus-9 AAACTTTTTAGTCCCTGTGTAATAAGTATTTGTGTCAATAAGCA
 ****************
Locus-44 AAACTTTTTAGTCCCTGTGTAATAAGTATTTGTATCAATAAGGC
 ****************
Locus-64 AAACTTTTTAGTCCCTGTGTAATAAGTATTTGTGTTAATAAGAT
 ****************
Locus-101 AAACTTTTTAGCCCCTGTGTAATAAGTATTTGTGTTAATAAGGT
 ****************
Locus-184 AAACTTTTTAGTCCCTGTGTAATAAGTATTTGTGTCAATAAGGT
 ****************
---------------------------------------------------------------------------------
SL-B1_Hvulgaris TGTGTAATAAG----------------------------------------------------
SL-F2_Hvulgaris ------------********AAAACACATCTAAACTTTTTTTAAGTATTTGTGTCAATAAG--
Locus-15 TGTGTAATAAGATATTACCGAAAACACATCTAAACTTTTTTTAAGTATTTGTGTCAATAAGCA
 *****************************************
---------------------------------------------------------------------------------
SL-B1_Hvulgaris TGTGTAATAAG------------------------------------------
SL-G_Hvulgaris -----------******************ACGTTAAATTTGTGTTAATAAG--
Locus-31 TGTGTAATAAGTTTTTTAGTATATAGTATACGTTAAATTTGTGTTAATAAGAT
 **********************
---------------------------------------------------------------------------------
SL-B1_Hvulgaris AAACTTTTTAGTCCCTGTGTAATAAG---------------------------------
SL-D_Hvulgaris **************************TTCTATTTGTGTTAATAAG--------------
SL-D/G_Hvulgaris ---------************************************TGTTAATAAG----
Locus-118 AAACTTTTTAGTCCCTGTGTAATAAGTTCTATTTGTGTTAATAAGTGTTAATAAGCA--
 *****************************
Locus-119 AAACTTTTTAATCCCTGTGTAATAAGTTCTATTTGTGTTAATAAGTGTTAATAAGCA--
 *****************************
---------------------------------------------------------------------------------
SL-F?_Hvulgaris **************************TATTTGTGTCAATAAG-----------------
SL-B1_Hvulgaris ---------*********************************TCCCTGTGTAATAAG—
Locus-148 AAACTTTTTAGTCCCTGTGTAATAAGTATTTGTGTCAATAAGTCCCTGTGTAATAAGGT
 *******************************
