# Supplementary material for: SLFinder, a pipeline for the novel identification of splice-leader sequences: a good enough solution for a complex problem
Source: BMC Bioinformatics. 2020 Jul 8;21:293. doi: 10.1186/s12859-020-03610-6 (PMC7346339; doi:10.1186/s12859-020-03610-6)
Supplement: Supplementary file 9 — Additional file 9: Supplementary Figure 3. Sequences of putative SL identified in H. vulgaris dataset. [file 12859_2020_3610_MOESM9_ESM.pptx]

## Slide 1
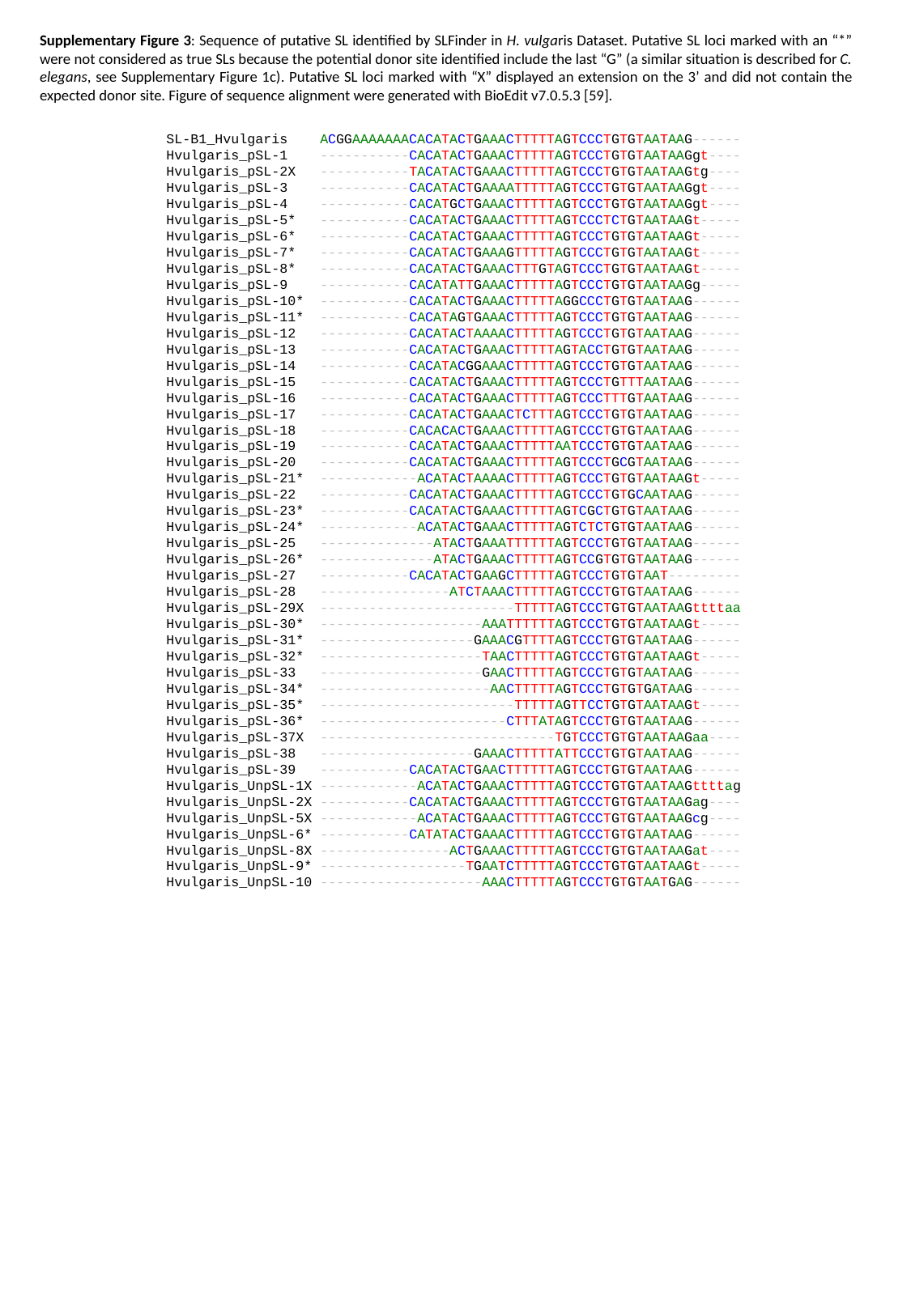

Supplementary Figure 3: Sequence of putative SL identified by SLFinder in H. vulgaris Dataset. Putative SL loci marked with an “*” were not considered as true SLs because the potential donor site identified include the last “G” (a similar situation is described for C. elegans, see Supplementary Figure 1c). Putative SL loci marked with “X” displayed an extension on the 3’ and did not contain the expected donor site. Figure of sequence alignment were generated with BioEdit v7.0.5.3 [59].
SL-B1_Hvulgaris ACGGAAAAAAACACATACTGAAACTTTTTAGTCCCTGTGTAATAAG------
Hvulgaris_pSL-1 -----------CACATACTGAAACTTTTTAGTCCCTGTGTAATAAGgt----
Hvulgaris_pSL-2X -----------TACATACTGAAACTTTTTAGTCCCTGTGTAATAAGtg----
Hvulgaris_pSL-3 -----------CACATACTGAAAATTTTTAGTCCCTGTGTAATAAGgt----
Hvulgaris_pSL-4 -----------CACATGCTGAAACTTTTTAGTCCCTGTGTAATAAGgt----
Hvulgaris_pSL-5* -----------CACATACTGAAACTTTTTAGTCCCTCTGTAATAAGt-----
Hvulgaris_pSL-6* -----------CACATACTGAAACTTTTTAGTCCCTGTGTAATAAGt-----
Hvulgaris_pSL-7* -----------CACATACTGAAAGTTTTTAGTCCCTGTGTAATAAGt-----
Hvulgaris_pSL-8* -----------CACATACTGAAACTTTGTAGTCCCTGTGTAATAAGt-----
Hvulgaris_pSL-9 -----------CACATATTGAAACTTTTTAGTCCCTGTGTAATAAGg-----
Hvulgaris_pSL-10* -----------CACATACTGAAACTTTTTAGGCCCTGTGTAATAAG------
Hvulgaris_pSL-11* -----------CACATAGTGAAACTTTTTAGTCCCTGTGTAATAAG------
Hvulgaris_pSL-12 -----------CACATACTAAAACTTTTTAGTCCCTGTGTAATAAG------
Hvulgaris_pSL-13 -----------CACATACTGAAACTTTTTAGTACCTGTGTAATAAG------
Hvulgaris_pSL-14 -----------CACATACGGAAACTTTTTAGTCCCTGTGTAATAAG------
Hvulgaris_pSL-15 -----------CACATACTGAAACTTTTTAGTCCCTGTTTAATAAG------
Hvulgaris_pSL-16 -----------CACATACTGAAACTTTTTAGTCCCTTTGTAATAAG------
Hvulgaris_pSL-17 -----------CACATACTGAAACTCTTTAGTCCCTGTGTAATAAG------
Hvulgaris_pSL-18 -----------CACACACTGAAACTTTTTAGTCCCTGTGTAATAAG------
Hvulgaris_pSL-19 -----------CACATACTGAAACTTTTTAATCCCTGTGTAATAAG------
Hvulgaris_pSL-20 -----------CACATACTGAAACTTTTTAGTCCCTGCGTAATAAG------
Hvulgaris_pSL-21* ------------ACATACTAAAACTTTTTAGTCCCTGTGTAATAAGt-----
Hvulgaris_pSL-22 -----------CACATACTGAAACTTTTTAGTCCCTGTGCAATAAG------
Hvulgaris_pSL-23* -----------CACATACTGAAACTTTTTAGTCGCTGTGTAATAAG------
Hvulgaris_pSL-24* ------------ACATACTGAAACTTTTTAGTCTCTGTGTAATAAG------
Hvulgaris_pSL-25 --------------ATACTGAAATTTTTTAGTCCCTGTGTAATAAG------
Hvulgaris_pSL-26* --------------ATACTGAAACTTTTTAGTCCGTGTGTAATAAG------
Hvulgaris_pSL-27 -----------CACATACTGAAGCTTTTTAGTCCCTGTGTAAT---------
Hvulgaris_pSL-28 ----------------ATCTAAACTTTTTAGTCCCTGTGTAATAAG------
Hvulgaris_pSL-29X ------------------------TTTTTAGTCCCTGTGTAATAAGttttaa
Hvulgaris_pSL-30* --------------------AAATTTTTTAGTCCCTGTGTAATAAGt-----
Hvulgaris_pSL-31* -------------------GAAACGTTTTAGTCCCTGTGTAATAAG------
Hvulgaris_pSL-32* --------------------TAACTTTTTAGTCCCTGTGTAATAAGt-----
Hvulgaris_pSL-33 --------------------GAACTTTTTAGTCCCTGTGTAATAAG------
Hvulgaris_pSL-34* ---------------------AACTTTTTAGTCCCTGTGTGATAAG------
Hvulgaris_pSL-35* ------------------------TTTTTAGTTCCTGTGTAATAAGt-----
Hvulgaris_pSL-36* -----------------------CTTTATAGTCCCTGTGTAATAAG------
Hvulgaris_pSL-37X -----------------------------TGTCCCTGTGTAATAAGaa----
Hvulgaris_pSL-38 -------------------GAAACTTTTTATTCCCTGTGTAATAAG------
Hvulgaris_pSL-39 -----------CACATACTGAACTTTTTTAGTCCCTGTGTAATAAG------
Hvulgaris_UnpSL-1X ------------ACATACTGAAACTTTTTAGTCCCTGTGTAATAAGttttag
Hvulgaris_UnpSL-2X -----------CACATACTGAAACTTTTTAGTCCCTGTGTAATAAGag----
Hvulgaris_UnpSL-5X ------------ACATACTGAAACTTTTTAGTCCCTGTGTAATAAGcg----
Hvulgaris_UnpSL-6* -----------CATATACTGAAACTTTTTAGTCCCTGTGTAATAAG------
Hvulgaris_UnpSL-8X ----------------ACTGAAACTTTTTAGTCCCTGTGTAATAAGat----
Hvulgaris_UnpSL-9* ------------------TGAATCTTTTTAGTCCCTGTGTAATAAGt-----
Hvulgaris_UnpSL-10 --------------------AAACTTTTTAGTCCCTGTGTAATGAG------
